# Trends in medications for autoimmune disorders during pregnancy and factors for their discontinuation: a population-based study

**DOI:** 10.1186/s12884-024-06932-y

**Published:** 2024-11-19

**Authors:** Sabine Mainbourg, Odile Sheehy, Jessica Gorgui, Evelyne Vinet, Anick Bérard

**Affiliations:** 1 Research Center, CHU Sainte Justine, 3175, Chemin de la Côte‑Sainte‑Catherine, Montreal, QC H3T 1C5 Canada; 2 Faculty of Pharmacy, University of Montreal, Montreal, QC Canada; 3 Laboratoire de Biométrie Et Biologie Evolutive, University of Claude Bernard Lyon1; CNRS, UMR 5558, Villeurbanne, 69622 France; 4 Faculty of Medicine, Divisions of Rheumatology & Clinical Epidemiology, McGill University Health Centre, Montreal, Canada; 5 Research Institute of the McGill University Health Centre, Montreal, Canada

**Keywords:** Pregnancy, Autoimmune disease, Inflammatory bowel disease, Rheumatoid arthritis, Spondyloarthropathies, Biologics

## Abstract

**Objectives:**

The medications used for autoimmune diseases have significantly evolved in recent years, but there is limited knowledge about how treatment practices changed during pregnancy. This study aimed to describe the temporal trends of immunosuppressants, immunomodulators and biologics use during pregnancy among women with autoimmune diseases, compare their use before, during, and after pregnancy, and identify factors predicting the discontinuation of these medications during pregnancy.

**Methods:**

Using data from the Quebec Pregnancy Cohort (1998–2015), which included women under the RAMQ prescription drug plan for at least 12 months before and after pregnancy, the analysis focused on those with at least one International Classification of Diseases Ninth or Tenth Revision code in the year before pregnancy for inflammatory bowel disease, rheumatoid arthritis, spondylarthropathies, connective tissue diseases, systemic lupus erythematosus, or vasculitis. Exposure to immunosuppressants, immunomodulators and biologics were evaluated before and during the pregnancy. Discontinuation during pregnancy was defined as having no prescriptions filled during pregnancy or overlapping with the first day of gestation (1DG), given that at least one prescription was filled in the year prior to pregnancy. Generalized estimating equations were applied to estimate adjusted odds ratios (aOR) for predicting medication discontinuation during pregnancy.

**Results:**

Among 441,570 pregnant women, 3,285 had autoimmune diseases. From 1998 to 2014, the use of immunomodulators increased from 3.7% to 11.9%, immunosuppressants from 4.1% to 13.7%, and biologics from 0% to 15.6%. During pregnancy, compared to before, there was a significant decrease in exposure to immunomodulators (8.6% to 5.4%), immunosuppressants (14.2% to 8.7%), and biologics (5.1% to 4.7%). Factors influencing discontinuation varied by medication type; for immunosuppressants, prior biologics use (aOR = 2.12, 95%CI 1.16–3.85) and the year of pregnancy (aOR = 0.93, 95%CI 0.89–0.98) were key factors, while for biologics, it was only the year of pregnancy (aOR = 0.68, 95%CI 0.54–0.86).

**Conclusions:**

The use of immunomodulators, immunosuppressants, and biologics has increased over time. However, exposure during pregnancy decreased, with recent years showing a lower rate of discontinuation. Understanding the factors influencing medication discontinuation during pregnancy can improve management strategies for women with autoimmune diseases.

## Introduction

Autoimmune rheumatic diseases primarily affect the joints and muscles [1] and encompass conditions such as systemic lupus erythematosus (SLE), other connective tissue diseases (CTD), rheumatoid arthritis (RA), and systemic vasculitis. Another group of autoimmune diseases—spondyloarthropathies (SpA)—predominantly impacts the axial skeleton and includes conditions like ankylosing spondylitis, psoriatic arthritis, and arthritis/spondylitis associated with inflammatory bowel disease (IBD) [2, 3]. Women of childbearing age are particularly affected by these autoimmune diseases [4–6], which are associated with a high prevalence of comorbidities [7–11], and the use of specific medications making pregnancy a critical period [12]. Deciding whether to continue or discontinue these medications during pregnancy is challenging [13–15], as disease control is crucial for maternal and fetal outcomes [12, 16, 17], but the medications themselves may pose risks [18–20]. The medications used for these diseases have evolved significantly in recent years. Initially, synthetic DMARDs were used, including immunosuppressants (thiopurines, methotrexate, mycophenolate mofetil), and immunomodulators (antimalarials, leflunomide, sulfasalazine). More recently, biologic DMARDs have been developed, predominantly TNF-α inhibitors, also known as biologics [21–23]. However, little is known about how their use has evolved during pregnancy.

This study aimed to 1) describe the temporal trends of immunosuppressants, immunomodulators and biologics use during pregnancy; 2) compare their use before, during, and after pregnancy among women with autoimmune diseases, and 3) identify factors predicting the discontinuation of these medications during pregnancy.

## Method

### Data source

We conducted a cohort study using the Quebec Pregnancy Cohort (QPC), which is described in detail elsewhere [24, 25]. In brief, the QPC is an ongoing, population-based cohort spanning from January 1, 1998 to December 31, 2015 with a prospective data collection concerning all pregnancies covered by the Régie de l’assurance maladie du Québec (RAMQ) Public Prescription Drug Insurance for at least 12 months before the first day of gestation and during pregnancy in the province of Quebec, Canada. This database includes detailed information on drug names, dosages, quantities dispensed, dispensation dates, treatment durations, and days supplied. It is linked with unique personal identifiers to other data sources: the RAMQ medical claims database for outpatient and inpatient diagnoses and socio-economic status, the Med-Echo database for in-hospital diagnoses, and the Quebec Institute of Statistics database for patient sociodemographic data.

Pregnant women are identified either through a prenatal visit or by a therapeutic procedure related to pregnancy recorded in RAMQ database or Med-Echo (e.g., ultrasound, amniocentesis, procedures related to planned or spontaneous abortion, delivery, etc.). The Med-Echo give the exact gestational age at the end of pregnancy, defined as the specific calendar date of a planned abortion, miscarriage, or delivery. Only clinically apparent or detected spontaneous and planned abortions are identified and reported here. The first day of gestation (1DG) was defined as the initial day of the last menstrual period, utilizing data pertaining to gestational age, a value validated through patient records and ultrasound results [26].

### Study population

Within the QPC, we identify women whose enrollment in the RAMQ drug plan was continuous for a further 12 months following the end of pregnancy. To be included, women were required to have at least one documented International Classification of Diseases Ninth or Tenth Revision (ICD-9 or ICD-10) code for an autoimmune disease in their outpatient or in-hospital diagnoses during the year preceding the 1DG. This predetermined list included IBD, which includes Crohn's disease and ulcerative colitis, RA, SpA, including ankylosing spondylitis and psoriatic arthritis, CTD/SLE, such as SLE, Sjögren's syndrome, systemic sclerosis, inflammatory myositis, other connective tissue diseases, and systemic vasculitis. The detailed list of the codes used is provided in Appendix 1. When a woman had multiple codes corresponding to different autoimmune diseases in the year preceding pregnancy, she was categorized into a specific group (multiple autoimmune diseases), in order to maintain mutually exclusive groups. The unit of analysis for this study was each individual pregnancy.

### Definition of medication use and periods of exposure

Within the medications covered by the RAMQ and dispensed by pharmacists, we identified filled prescriptions for medications specific to autoimmune diseases, including immunomodulators, immunosuppressants, and biologics using their corresponding Quebec generic codes. The complete list of codes is available in Appendix 2. In summary, immunomodulators included dapsone, hydroxychloroquine, leflunomide, minocycline, penicillamine, and sulfasalazine. Immunosuppressants included thiopurines (azathioprine and 6-mercaptopurine), mycophenolic acid, and methotrexate. Biologics included TNF-α inhibitors (adalimumab, certolizumab, etanercept, golimumab, and infliximab), anakinra, ustekinumab, tocilizumab, abatacept, and belimumab.

We examined the prevalence of dispensed medication within three distinct study intervals: 1) before pregnancy, defined as the year before the 1DG; 2) during pregnancy, from the 1DG to the end of the pregnancy, regardless of the pregnancy outcome; and 3) after pregnancy, defined as the year after the end of the pregnancy. A 1-year period before pregnancy was selected to account for the prolonged time to conception and reduced fecundity in women with autoimmune diseases [27], and one year after pregnancy for consistency. Medication use for each period of interest was defined as having at least one prescription filled during the relevant period (i.e. before, during or after pregnancy) or a prescription filled before the period but with duration overlapping with the first day of that period. Prescriptions of methotrexate filled during the first trimester with a dose exceeding 25 mg/week were excluded from the analysis, as this medication can also be used as an abortifacient during this period at higher doses than those employed in the treatment of autoimmune diseases. For each category of medication, we defined its discontinuation during pregnancy as the absence of any prescriptions filled during pregnancy or overlapping with the 1DG, provided that at least one prescription had been filled in the year before pregnancy.

### Determinants of specific medication discontinuation

To evaluate potential factors influencing the discontinuation of medication during pregnancy, we took into account the following variables: 1) socio-demographic characteristics: age at the 1DG, welfare recipient (yes/no), and rural dweller (yes/no); 2) maternal chronic comorbidities measured in the year before pregnancy: hypertension, diabetes mellitus, asthma, depression or anxiety disorder and thyroid disorder defined by at least one ICD-9, ICD-10 code or at least one prescription filled for one of these conditions (Appendix 3); 3) healthcare utilization, defined by the number of visits in the year before pregnancy to a general practitioner (GP) or to a specialist (e.g. immunologist, nephrologist, pulmonologist, gastroenterologist, neurologist, rheumatologist, dermatologist, internist), and emergency visits and/or hospitalizations in the year before pregnancy (yes/no); 4) use of immunomodulators, immunosuppressants or biologics before pregnancy; and 6) type of autoimmune disease.

### Statistical analysis

Descriptive statistics were used to summarize the characteristics of the study cohort. To assess temporal trends in medication use, we employed linear regression models. For each specific time period under consideration, prescribed medication prevalence was calculated as the proportion of pregnancies exposed during that period, computed for all pregnancies in the given time period, both across the entire study cohort and stratified by autoimmune diseases. To compare the prevalence of use before pregnancy with the prevalence of use during and after pregnancy, we utilized McNemar's test. We examined factors influencing specific medication discontinuation through univariate and multivariate logistic regression models. For the multivariate analysis, we used generalized estimating equations (GEEs) to account for the correlation between multiple pregnancies for the same woman, recognizing that the responses may be related. GEEs provide a robust way to estimate the average effects while accounting for the within-subject correlation by using an independence working correlation structure, which enhances the validity of our results. The results were presented using odds ratios (OR) for univariate analysis and adjusted odds ratios (aOR) for multivariate analysis with their 95% confidence interval (CI). All analyses were two-tailed, and statistical significance was set at *p* ≤ 0.05. We conducted these analyses using R version 4.8.1 (R Language and Environment for Statistical Computing, Vienna, Austria) [28].

### Ethical considerations

Our study was approved by the CHU Sainte-Justine Institutional Review Board. Additionally, database linkages were authorized by the Quebec Commission d’Accès à l’information.

## Results

**Fig. 1:**
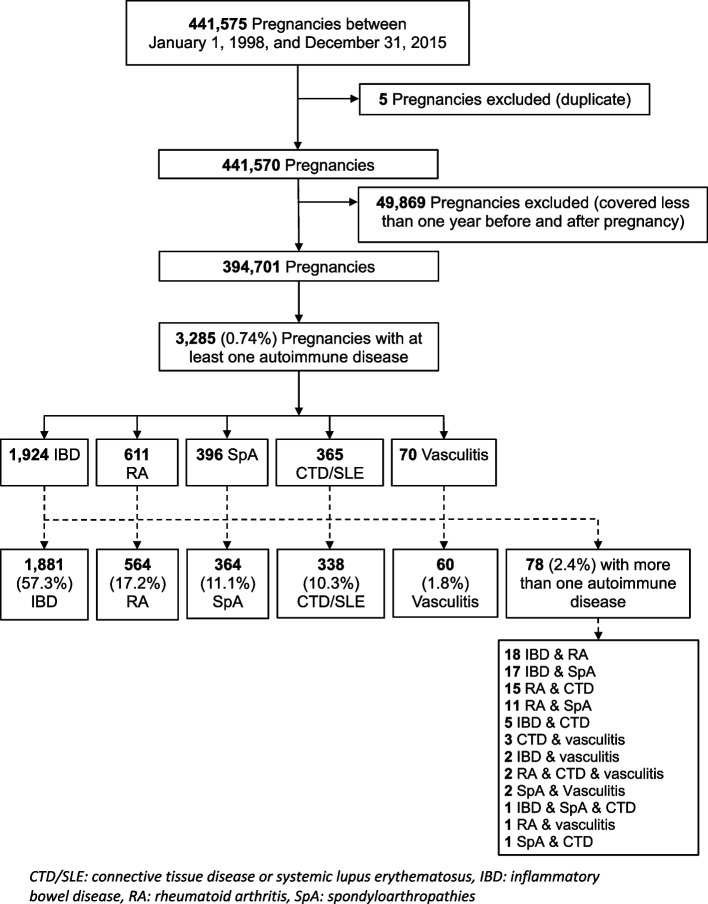
Flow chart of our cohort study

Out of 441,570 pregnancies within the cohort, 3,285 (0.74%) were identified as having at least one autoimmune disease in the year before pregnancy. The overall mean gestational duration was 28.5 weeks (± SD 11.8). Of the pregnancies, 57.2% (1,880) ended in delivery (live or stillborn), 34.5% (1,134) in planned/ induced abortion, and 8.2% (271) in spontaneous abortion. The most prevalent autoimmune condition observed was IBD, accounting for 57.3% of cases, followed by RA at 17.2%, SpA at 11.1%, CTD/SLE at 10.3%, and vasculitis at 1.8%. Furthermore, 2.4% of pregnancies were associated with multiple autoimmune diseases, with RA being a component in 60.3% of these cases (Fig. 1).

SLE women diagnosed with RA or CTD/SLE were older at the 1DG compared to those with IBD and vasculitis. Women affected by multiple autoimmune diseases displayed a higher frequency of visits to the GP and specialists in the year before pregnancy. They also had more frequent visits at emergency departments and hospitals in the year before pregnancy. Women with CTD/SLE had a higher prevalence of chronic hypertension, while those with vasculitis exhibited a greater occurrence of diabetes. Additionally, women affected by multiple autoimmune diseases showed a higher prevalence of thyroid disorders. However, the prevalence of asthma, depression or anxiety disorders did not differ significantly among the specific autoimmune diseases (Table 1).

**Table 1:** Characteristics of the study cohort and of each autoimmune disease in mutually exclusive groups in the year before pregnancy

Characteristic	Any autoimmune disease *n* = 3,285	IBD *n* = 1,881	RA *n* = 564	SpA *n* = 364	CTD/ SLE *n* = 338	Vasculitis *n* = 60	Multiple AD *n* = 78
Maternal age at the 1DG, years, mean (sd)	28.8 (6.0)	28.2 (5.8)	29.9 (6.4)	29.4 (6.1)	29.9 (5.8)	28.3 (5.7)	29.5 (6.1)
Duration of gestation, weeks, mean (sd)	28.5 (11.8)	28.6 (11.8)	29.5 (11.6)	27.9 (11.9)	27.3 (11.8)	26.2 (12.4)	30.0 (10.8)
Welfare recipients, *n* (%)	916 (27.9)	498 (26.5)	175 (31.0)	102 (28.0)	97 (28.7)	22 (36.7)	22 (28.2)
Rural dweller (vs urban), *n* (%)	2,754 (83.8)	1,550 (82.4)	479 (84.9)	306 (84.1)	305 (90.2)	48 (80.0)	66 (84.6)
Number of GP visits, mean (sd)	10.2 (14.5)	11.0 (13.7)	8.5 (9.2)	9.8 (14.8)	6.6 (7.4)	12.9 (25.0)	19.4 (42.3)
Number of specialist physician^1^ visits, mean (sd)	0.5 (1.7)	0.4 (1.2)	0.3 (0.8)	0.3 (0.7)	0.8 (3.7)	1.0 (2.1)	1.5 (2.6)
Emergency visit or hospitalization, *n* (%)	858 (26.1)	563 (29.9)	104 (18.4)	56 (15.4)	82 (24.3)	22 (36.7)	31 (39.7)
Chronic hypertension, *n* (%)	183 (5.6)	65 (3.5)	32 (5.7)	21 (5.8)	55 (16.3)	3 (5.0)	7 (9.0)
Diabetes mellitus, *n* (%)	81 (2.5)	34 (1.8)	18 (3.2)	9 (2.5)	12 (3.6)	5 (8.3)	3 (3.8)
Asthma, *n* (%)	541 (16.5)	310 (16.5)	83 (14.7)	56 (15.4)	61 (18.0)	16 (26.7)	15 (19.2)
Depression or anxiety disorder, *n* (%)	791 (24.1)	459 (24.4)	128 (22.7)	96 (26.4)	76 (22.5)	11 (18.3)	21 (26.9)
Thyroid disorder, *n* (%)	168 (5.1)	73 (3.9)	49 (8.7)	20 (5.5)	19 (5.6)	0 (0.0)	7 (9.0)
Exposure to medication before pregnancy, *n* (%)							
Immunomodulators	282 (8.6)	40 (2.1)	111 (19.7)	12 (3.3)	99 (29.3)	2 (3.3)	18 (23.1)
Immunosuppressants	467 (14.2)	311 (16.5)	72 (12.8)	12 (3.3)	41 (12.1)	4 (6.7)	27 (34.6)
Biologics	169 (5.1)	108 (5.7)	30 (5.3)	16 (4.4)	3 (0.9)	1 (1.7)	11 (14.1)

*1DG* first day of gestation, *AD* autoimmune disease, *CTD* connective tissue disease, *GP* general practitioner, *IBD* inflammatory bowel disease, *RA* rheumatoid arthritis; sd: standard deviation, *SLE* systemic lupus erythematosus, SpA: spondyloarthritis

^1^Immunologist, nephrologist, pulmonologist, gastroenterologist, neurologist, rheumatologist, dermatologist, internist

^2^denominator *N* included all pregnancies with duration of gestation ≥ 20 weeks for gestational diabetes and gestational hypertension

Before pregnancy, the study population exhibited an 8.6% exposure prevalence to immunomodulators, with the highest prevalence observed among women with CTD/SLE (29.3%) and multiple autoimmune diseases (23.1%). Exposure to immunosuppressants was noted in 14.2% of the study population, with the highest prevalence observed among women with multiple autoimmune diseases (34.6%). Biologics exposure accounted for 5.1% within the study population, with the highest prevalence observed among women with multiple autoimmune diseases (14.1%, Table 1).

### Trends in prevalence of medication use during pregnancy

**Fig. 2:**
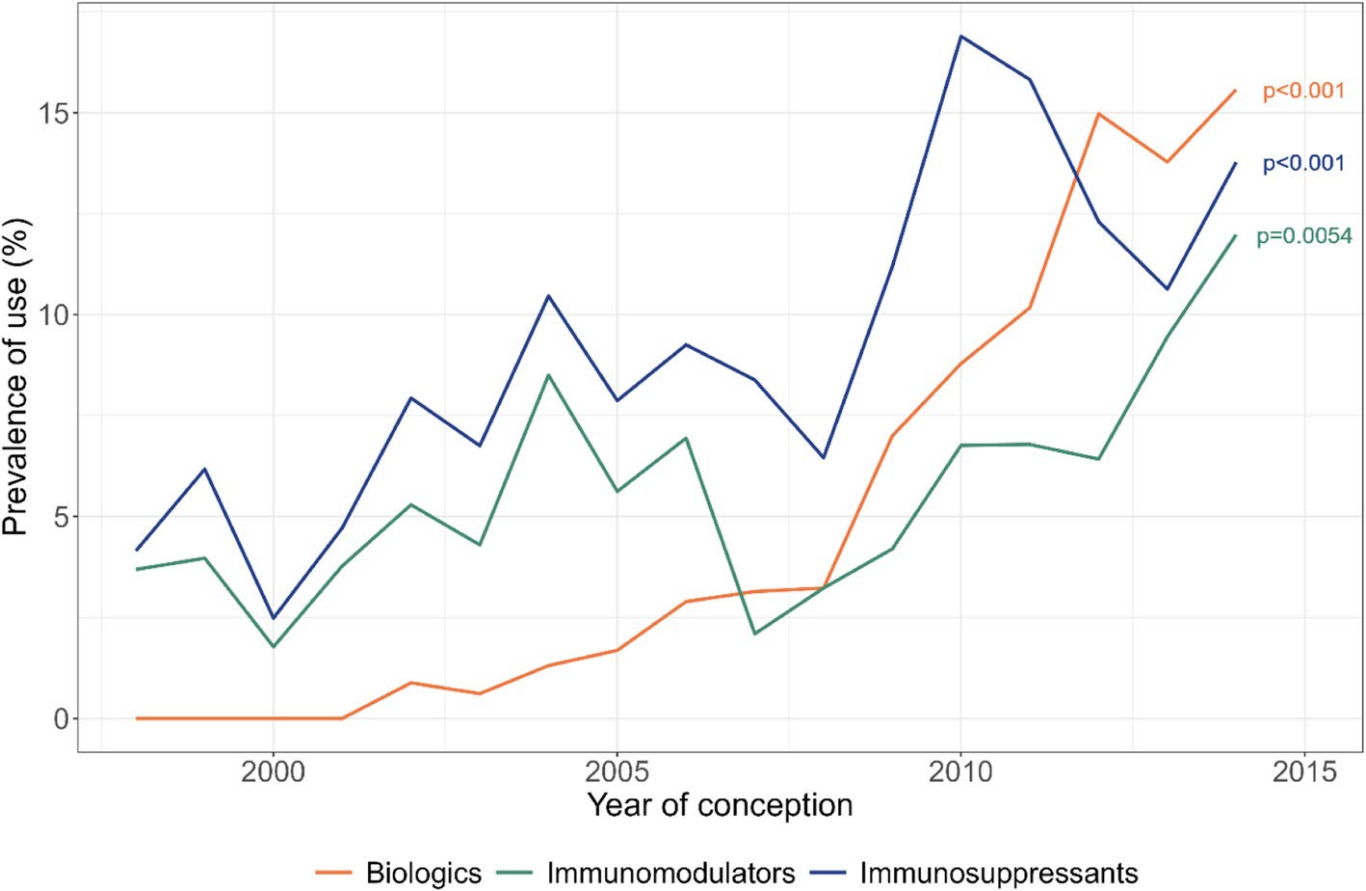
Evolution of prevalence of use of specific medications at any time in pregnancy from 1998 to 2014. Biologics include TNF-α inhibitors (adalimumab, certolizumab, etanercept, golimumab, and infliximab), anakinra, ustekinumab, tocilizumab, abatacept, and belimumab; immunomodulators include dapsone, hydroxychloroquine, leflunomide, minocycline, penicillamine, and sulfasalazine; immunosuppressants include thiopurines (azathioprine and 6-mercaptopurine), mycophenolic acid, and methotrexate

The prevalence of immunomodulators and immunosuppressants use during pregnancy increased by threefold between 1998 and 2014. Immunomodulator use significantly increased from 3.7% in 1998 to 11.9% in 2014 (*p* = 0.0054), while immunosuppressant use rose from 4.1% in 1998 to 13.7% in 2014 (*p* < 0.001). The use of biologics was first identified in 2002. There was a 19-fold increase in the prevalence of their use rising from 0.8% in 2002 to 15.6% in 2014 (*p* < 0.001). The prevalence trends in biologics use exhibited a two-part division with distinct slopes: an initial increase from 2002 to 2008 at a rate of + 0.4% per year, followed by a more substantial increase from 2008 to 2015 at a rate of + 2.1% per year. The prevalence of biologics use exceeded that of immunomodulators in 2007 and that of immunosuppressants in 2012 (Fig. 2).

### Medication use before, during and after pregnancy

**Fig. 3:**
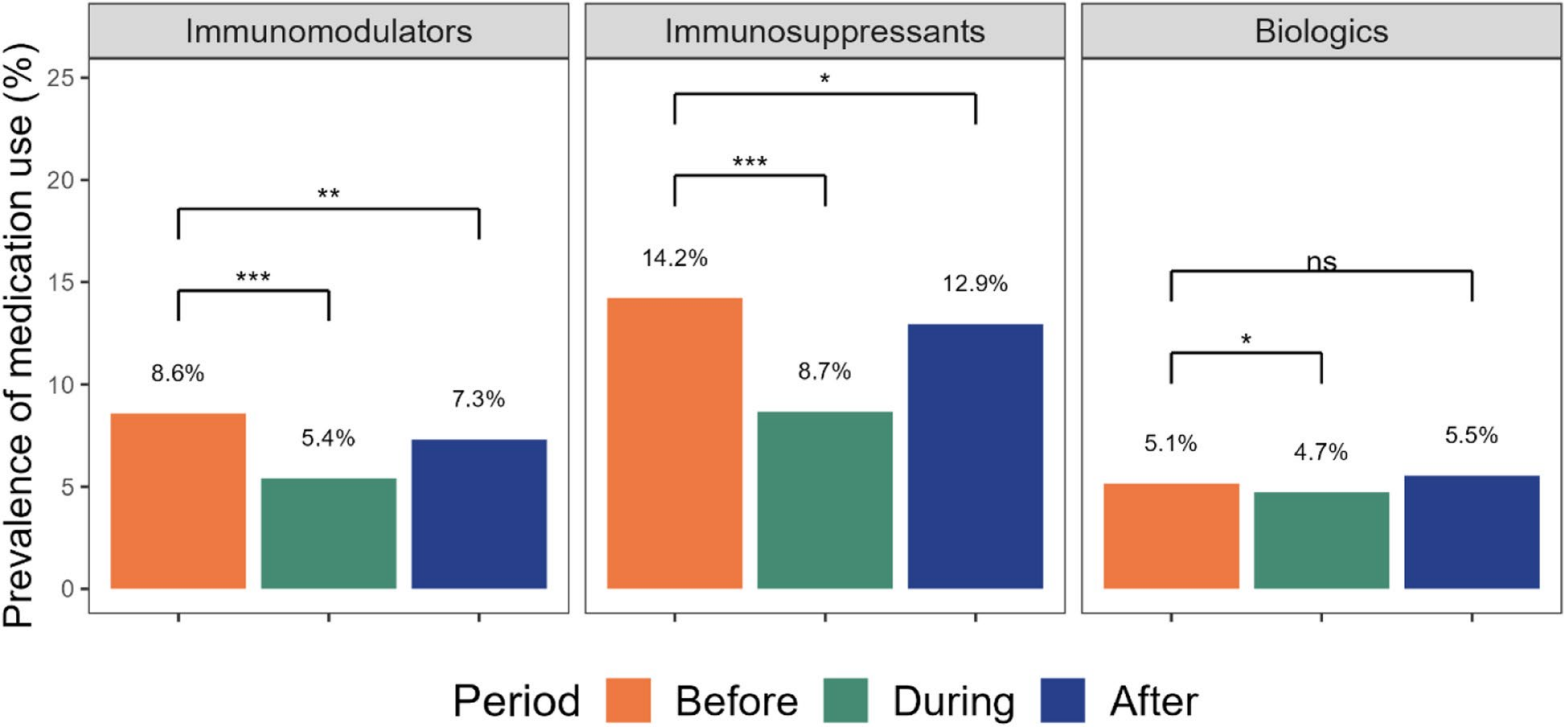
Prevalence of medication use in pregnancies with autoimmune diseases, before pregnancy (in the year before the 1st day of gestation), during pregnancy, and after pregnancy (in the year after the end of pregnancy). Biologics include TNF-α inhibitors (adalimumab, certolizumab, etanercept, golimumab, and infliximab), anakinra, ustekinumab, tocilizumab, abatacept, and belimumab; immunomodulators include dapsone, hydroxychloroquine, leflunomide, minocycline, penicillamine, and sulfasalazine; immunosuppressants include thiopurines (azathioprine and 6-mercaptopurine), mycophenolic acid, and methotrexate. The level of statistical significance is represented by stars (***: *p*-value < 0.001, **: *p*-value < 0.01, *: *p*-value < 0.05, ns: non-significant)

Figure 3 presents the trends for the three periods. The prevalence of immunomodulators use was 8.6% in the year before pregnancy and significantly reduced to 5.4% during pregnancy (*p* < 0.001). In the year following pregnancy, the prevalence of immunomodulators use increased to 7.3%, although it remained significantly lower than the level before pregnancy (*p* = 0.0037, Fig. 3). In the case of immunosuppressants, their prevalence of use was 14.2% in the year before pregnancy and it significantly declined to 8.7% during pregnancy (*p* < 0.001). After pregnancy, the prevalence of immunosuppressants use was 12.9%, which was significantly lower compared to before pregnancy (*p* = 0.011). The prevalence of biologics use was 5.1% in the year before pregnancy and decreased to 4.7% during pregnancy (*p* = 0.011). In the year following pregnancy, the prevalence of biologics use increased to 5.5%, although this increase was not statistically significant compared to the period before pregnancy (*p* = 0.14).

### Medication use according to autoimmune disease type

**Fig. 4:**
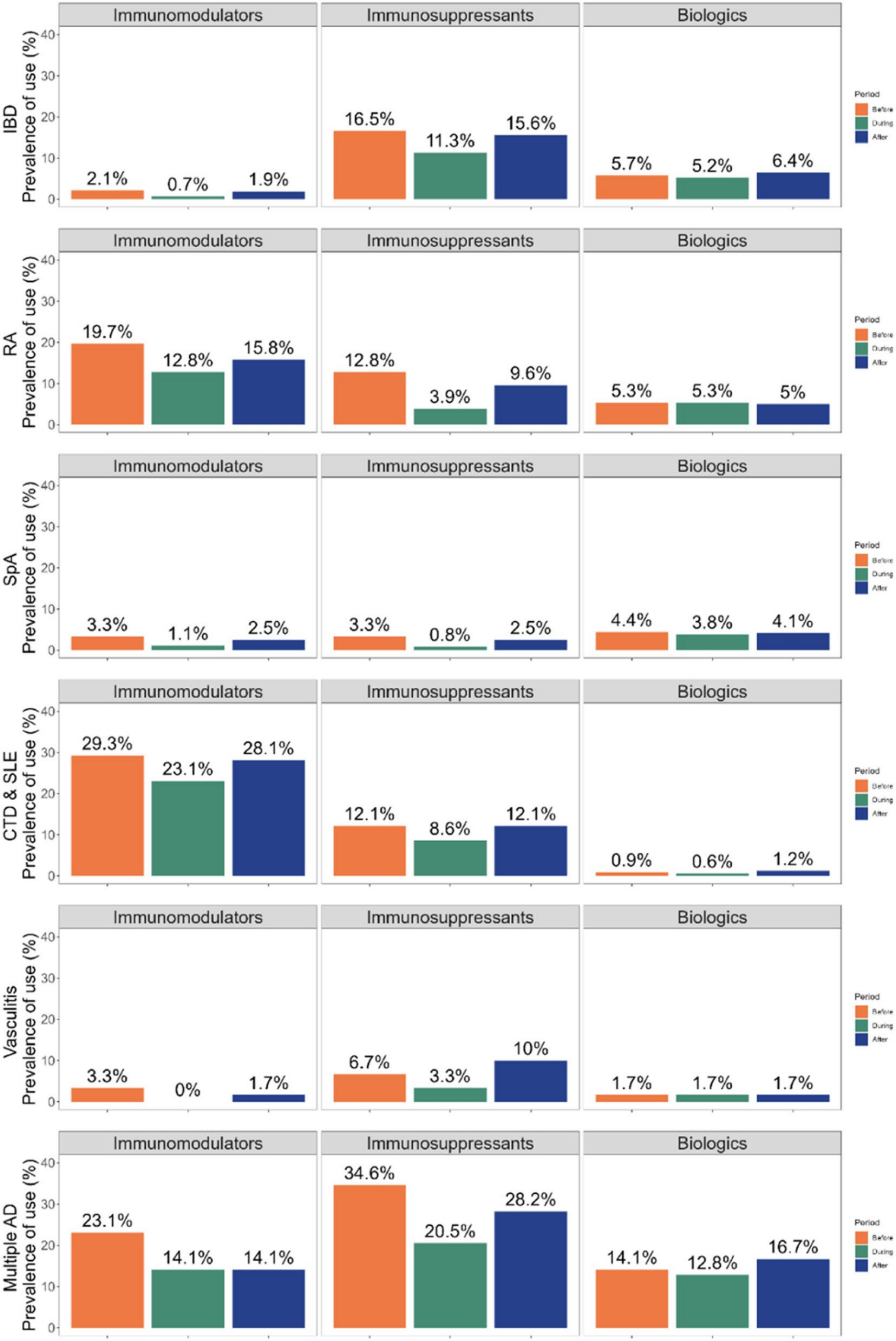
Prevalence of use of immunomodulators, immunosuppressants and biologics in the year before the 1st day of gestation, during pregnancy, and in the year after pregnancy according to specific autoimmune disease. Immunomodulators include dapsone, hydroxychloroquine, leflunomide, minocycline, penicillamine, and sulfasalazine; immunosuppressants include thiopurines (azathioprine and 6-mercaptopurine), mycophenolic acid, and methotrexate; biologics include TNF-α inhibitors (adalimumab, certolizumab, etanercept, golimumab, and infliximab), anakinra, ustekinumab, tocilizumab, abatacept, and belimumab. IBD: Inflammatory disease disease, RA: rheumatoid arthritis, SpA: spondyloarthropathies, CTD & SLE: connective tissue disease or systemic lupus erythematosus, AD: autoimmune disease

The prevalence of immunomodulators use was highest in pregnancies associated with CTD/SLE (29.3% before pregnancy, 23.1% during pregnancy, 28.1% after pregnancy). This was followed by pregnancies affected by multiple autoimmune diseases (23.1% before pregnancy, 14.1% during pregnancy, 14.1% after pregnancy), and those with RA (19.7% before pregnancy, 12.8% during pregnancy, 15.8% after pregnancy). In the case of immunosuppressants use, it was more than two times higher in pregnancies affected by multiple autoimmune diseases (34.6% before pregnancy, 20.5% during pregnancy, 28.2% after pregnancy) compared to other conditions (ranging from 16.5% to 6.7% before pregnancy, 11.3% to 0.8% during pregnancy, and 15.6% to 2.5% after pregnancy). The prevalence of biologics use was approximately three times higher in pregnancies with multiple autoimmune diseases (14.1% before pregnancy, 12.8% during pregnancy, 16.7% after pregnancy) compared to other conditions (ranging from 5.7% to 0.9% before pregnancy, 5.3% to 0.6% during pregnancy, and 6.4% to 1.2% after pregnancy) (Fig. 4).

### Predictors of specific medication discontinuation

#### Immunomodulators

Among the 282 pregnant women exposed to immunomodulators before pregnancy, 42.9% (*n* = 121) discontinued their use during pregnancy. In the multivariate analysis, the older the women were at the onset of their pregnancy, the less likely they were to discontinue their immunomodulators (aOR = 0.94, 95% CI 0.89–0.99). Using multiple autoimmune diseases as the reference group, immunomodulator discontinuation was positively associated with IBD (aOR = 9.66, 95% CI 2.50–37.35) and SpA (aOR = 7.63, 95% CI 1.46–39.71).

#### Immunosuppressants

Among 467 pregnant women who took immunosuppressants before pregnancy, 196 (39.4%) discontinued their treatment during pregnancy. In the multivariate analysis, the older the women were at the onset of their pregnancy, the less likely they were to discontinue their immunomodulators (aOR = 0.95, 95% CI 0.91–0.99). Discontinuation was negatively associated with welfare recipient status (aOR = 0.58, 95% CI 0.35–0.96) and with time, pregnant women were less likely to discontinue their immunosuppressants (aOR = 0.93 for each increase of one calendar year, 95% CI 0.89–0.98). In addition, immunosuppressant discontinuation was positively associated with biologics use before pregnancy (aOR = 2.12, 95% CI 1.16–3.85). Women with RA were more likely to discontinue the use of immunosuppressants during pregnancy when compared to women with multiple autoimmune diseases (aOR = 3.43, 95% CI 1.23–9.61).

#### Biologics

Among 169 pregnant women exposed to biologics before pregnancy, 20 (11.8%) discontinued their treatment during pregnancy. Due to small sample size, adjustments for autoimmune disease and diabetes mellitus were not feasible. With time, pregnant women were less likely to discontinue their biologics in the multivariate analysis (aOR = 0.68 for each increase of one calendar year, 95% CI 0.54–0.86) (Table 2).

**Table 2:** Multivariate logistic regression models assessing determinants of medication discontinuation at the first trimester of pregnancy Data are presented as OR (95% CI). Biologics include TNF-α inhibitors (adalimumab, certolizumab, etanercept, golimumab, and infliximab), anakinra, ustekinumab, tocilizumab, abatacept, and belimumab; immunomodulators include dapsone, hydroxychloroquine, leflunomide, minocycline, penicillamine, and sulfasalazine; immunosuppressants include thiopurines (azathioprine and 6-mercaptopurine), mycophenolic acid, and methotrexate

	**Immunomodulators**		**Immunosuppressants**		**Biologics**	
	**OR**	**adjusted OR**	**OR**	**adjusted OR**	**OR**	**adjusted OR**
Maternal age at the 1DG	0.96 (0.92-1.00)	0.94 (0.89-0.99)	0.97 (0.94-1.01)	0.95 (0.91-0.99)	0.99 (0.92-1.06)	1.00 (0.91-1.09)
Welfare recipients	1.00 (0.59-1.70)	0.81 (0.42-1.54)	0.64 (0.41-0.98)	0.58 (0.35-0.96)	1.10 (0.38-3.16)	0.60 (0.17-2.06)
Rural dweller (vs urban)	0.83 (0.43-1.63)	1.11 (0.50-2.45)	0.84 (0.51-1.40)	0.85 (0.51-1.42)	2.19 (0.46-10.38)	5.27 (0.68-40.61)
Number of GP visits	1.04 (1.01-1.07)	1.01 (0.98-1.04)	1.00 (0.99-1.01)	1.00 (0.99-1.02)	1.03 (1.00-1.06)	1.02 (0.99-1.06)
Number of specialist physician visits	1.18 (0.97-1.44)	1.26 (0.97-1.64)	0.89 (0.79-1.00)	0.83 (0.68-1.02)	1.00 (0.84-1.20)	0.93 (0.71-1.22)
Emergency visit or hospitalization	0.97 (0.51-1.83)	0.86 (0.39-1.88)	0.94 (0.62-1.41)	1.23 (0.78-1.93)	2.03 (0.81-5.07)	1.80 (0.55-5.83)
Chronic hypertension	0.95 (0.45-2.03)	1.46 (0.57-3.77)	0.95 (0.48-1.88)	1.21 (0.55-2.68)	3.45 (1.01-11.81)	3.30 (0.78-13.96)
Diabetes mellitus	0.67 (0.20-2.16)	1.08 (0.30-3.90)	2.37 (0.87-6.52)	2.26 (0.89-5.79)	ꟷ	ꟷ
Asthma	1.41 (0.78-2.55)	1.14 (0.57-2.26)	1.17 (0.71-1.93)	1.47 (0.84-2.56)	0.59 (0.16-2.19)	0.30 (0.07-1.27)
Depression or anxiety disorder	1.60 (0.91-2.83)	1.32 (0.67-2.64)	0.87 (0.57-1.34)	0.89 (0.52-1.52)	1.43 (0.50-4.08)	1.58 (0.51-4.91)
Thyroid disorder	0.69 (0.28-1.71)	0.89 (0.30-2.59)	1.32 (0.55-3.15)	1.07 (0.46-2.47)	1.94 (0.42-8.95)	3.49 (0.55-22.04)
Autoimmune diagnosis						
Multiple AD	Reference	Reference	Reference	Reference	ꟷ	ꟷ
IBD	6.29 (1.95-20.31)	9.66 (2.50-37.35)	0.80 (0.35-1.79)	0.67 (0.27-1.67)	ꟷ	ꟷ
RA	1.11 (0.42-2.98)	1.81 (0.53-6.17)	3.53 (1.40-8.94)	3.43 (1.23-9.61)	ꟷ	ꟷ
SpA	4.71 (1.04-21.29)	7.63 (1.46-39.71)	4.36 (0.93-20.53)	3.96 (0.84-18.63)	ꟷ	ꟷ
CTD/SLE	0.53 (0.19-1.45)	0.71 (0.20-2.54)	0.60 (0.21-1.69)	0.57 (0.19-1.71)	ꟷ	ꟷ
Vasculitis	ꟷ	ꟷ	4.36 (0.35-54.94)	4.59 (0.28-74.84)	ꟷ	ꟷ
Medication use before pregnancy						
Immunomodulators	ꟷ	ꟷ	1.83 (1.06-3.15)	1.27 (0.60-2.66)	1.19 (0.44-3.23)	0.70 (0.25-2.00)
Immunosuppressants	0.68 (0.39-1.18)	0.71 (0.36-1.37)	ꟷ	ꟷ	1.70 (0.48-5.98)	2.17 (0.55-8.59)
Biologics	1.85 (0.81-4.22)	1.67 (0.65-4.31)	1.53 (0.94-2.51)	2.12 (1.16-3.85)	ꟷ	ꟷ
Calendar year of 1DG	0.99 (0.94-1.03)	1.02 (0.96-1.08)	0.97 (0.93-1.01)	0.93 (0.89-0.98)	0.74 (0.64-0.87)	0.68 (0.54-0.86)

*1DG*: first day of gestation; *AD*: autoimmune disease;* CTD*: connective tissue disease;* GP*: general practitioner,* IBD*: inflammatory bowel disease;* OR*: odds ratio;* RA*: rheumatoid arthritis; *sd*: standard deviation;* SLE*: systemic lupus erythematosus;* SpA*: spondyloarthritis

## Discussion

Our findings indicate a rising trend in immunomodulators, biologics and immunosuppressants use among pregnant women with autoimmune diseases between 1998 and 2014. The observed upward trend in biologics use during pregnancy aligns with the findings reported in various epidemiological studies [15, 29, 30] conducted from 2002 to 2012. This trend can be attributed to the increasing prevalence of biologics use among the patients suffering from autoimmune diseases [31], the paramount significance of disease control [17], and the improved awareness and understanding of their safety profiles during pregnancy [32–34]. The first biologics (etanercept and infliximab) entered the Canadian market in 2000 [35], explaining the beginning of their use during pregnancy in 2002. Furthermore, the significant increase in their use after 2008 as evident in our findings and also reported in a study conducted in British Columbia [29], can largely be attributed to the introduction of the first biosimilar in Canada in 2009. This introduction led to reduced prices, thereby facilitating the widespread adoption of these medications for the management of autoimmune conditions.

The prevalence of biologics use during pregnancy varies across countries; for instance, in 2012 it ranged from 8 to 15% [15, 29]. These differences may reflect disparities in access to these costly medications and variations in clinicians' prescribing confidence during pregnancy. We also observed a rising trend in the use of immunosuppressants and immunomodulators, particularly hydroxychloroquine, used in pregnant patients with SLE, as supported by literature since the early 2000s [36–38]. However, some studies have shown stable or slightly reduced use of immunomodulators or immunosuppressants for IBD or rheumatic diseases [15, 30].

For immunomodulators, immunosuppressants and biologics, we observed a decreased prevalence of use during pregnancy compared to before, as previously described [15, 29, 39]. Among women treated before pregnancy, approximately half were not exposed to immunomodulators, a third to immunosuppressants, and an eighth to biologics during pregnancy. This may be due to medications contraindicated during pregnancy, preconception counseling to start pregnancy in a disease-quiescent state [40, 41], and concerns about potential fetal risks despite available safety data. Our multivariate analysis revealed an association between the year of the 1DG and a decreased likelihood of discontinuation, significant for immunosuppressants and biologics. This suggests that a tangible shift in clinical practice has occurred between 1998 and 2014. It would be interesting to correlate these trends with corticosteroid use during pregnancy, but it is challenging in an administrative database to distinguish between corticosteroid prescriptions for autoimmune diseases and those for other conditions.

Our study had several strengths. First, we used a population-based design with a large sample of pregnant women, notable given the rarity of autoimmune diseases. The 16-year dataset captured prescription trends for immunomodulators, immunosuppressants, and biologics, allowing us to track the introduction of biologics. This extensive duration enhances the suitability of our study for delineating secular trends. We adjusted our model for the calendar year of the 1DG, which allowed us to account for changes in population structure over time. The QPC data is recorded prospectively, providing accurate and detailed medication histories during pregnancy, free from recall bias.

There are also limitations. Biologics use may be underestimated due to intravenous biologics administered during hospitals stays, which are not captured in our data. Our classification combined drugs whose use was either approved (for example thiopurine) or non-approved (for example methotrexate) during pregnancy, which may have led to variability in prescription prevalence within categories, reflecting disease severity and drug switches during pregnancy. The broad definition of discontinuation, though potentially over-inclusive, is supported by the specificity of these mediations for severe diseases, suggesting that even a single prescription fill is significant. Misclassification risk exists, particularly for short exposures in early pregnancy when medication may have been discontinued after discovery of pregnancy.

Medication use estimates may be inaccurate, as they were based on prescription fills, which may not match actual consumption. However, previous research by our team showed strong validity between dispensation data and actual medication use during pregnancy with positive and negative predictive values above 80% [42]. Early miscarriages may also have led to underestimation of use during pregnancy. Despite our sample size was large, some autoimmune diseases like vasculitis were underrepresented, reducing statistical power. We used a sensitive case definition with one outpatient diagnosis, but we limited diagnoses to the year before pregnancy and focused on discontinuation among prior users, improving diagnostic accuracy for this subpopulation as the medications under consideration are specifically associated with the diseases included. Lastly, our data only extends to 2015, so it may not reflect current usage trends.

## Conclusion

Our study reveals a progressive rise in the utilization of biologics, immunosuppressants, and immunomodulators during pregnancy among women with autoimmune diseases from 1998 to 2015. Notably, the discontinuation of these medications during pregnancy was highest for immunomodulators, followed by immunosuppressants and biologics, with a declining trend observed in recent years.

## Data Availability

No datasets were generated or analysed during the current study.

## Appendix 1

List of ICD-9 and ICD-10 diagnosis codes used for autoimmune diagnosis

**Table:**

**Diseases**	**ICD-9**	**ICD-10**
**Inflammatory bowel disease**		
Crohn’s disease	555.x	K50, M07.4
Ulcerative colitis	556.x	K51, M07.5
**Rheumatoid arthritis**	714.x	M05.x, M06.0, M06.2, M06.3, M06.8, M06.9, M08.0
**Connective tissue diseases**	710	
Systemic lupus erythematosus	710; 695.4	M32.1 ; M32.8 ; M32.9
Sjogren syndrome (sicca syndrome)	710.2	M35.0
Systemic sclerosis	710.1	M34; M34.1 ; M34.8 ; M34.9
Inflammatory myositis	710.3 ; 710.4	M33; M33.1 ; M33.2 ; M33.9
Other connective tissue disease	710.8 ; 710.9	M36.8 ; M35.1 ; M35.9
**SpA**		
Axial spondyloarthritis	720.x	M45, M46.1, M46.8, M46.9, M08.1
Psoriatic arthritis	696.0	M07.0–M07.3,M07.6, L40.5
**Systemic vasculitis**		
Polyarteritis nodosa	446.0, 446.9	M30; M30.8
Granulomatosis with polyangiitis	446.4	M31.3
Microscopic polyangiitis		M31.7
Eosinophilic granulomatosis with polyangiitis		M30.1
Cryoglobulinemia		D89.1
Behçet disease	136.1 ; 711.2	M35.2
Takayasu’s disease	446.7	M31.4
IgA vasculitis (Henoch-Schonlein purpura)	287.0	D69.0

## Appendix 2

List of medications of interest

**Table:**

**Generic name**	**Quebec generic code**
**Immunomodulators**	
Leflunomide	46649, 47362
Sulfasalazine	45420
Hydroxychloroquine	04654
Dapsone	02431
Penicillamine	06994
Minocycline	6279
**Immunosuppressants**	
Azathioprine	00754, 37820
Mercaptopurine	05733
Mycophenolic acid	47157, 47559
Methotrexate	47651
**Biologics**	
Etanercept	46711, 47438
Infliximab	48034, 46739
Adalimumab	47522
Golimumab	47798
Certolizumab	47796, 47968
Tocilizumab	47841
Anakinra	46829
Ustekinumab	47757
Belimumab	47872
Abatacept	47618

## Appendix 3

### List of ICD-9 and ICD-10 diagnosis codes and medications used for the comorbidities

#### Hypertension

- ICD-9 codes: 401.0-405.9, 642.0-642.2,642.7
- ICD-10 codes: I10, I11, I12, I13, I15, I67.4, O10-O11, O16
- Medication generic codes:

**Table:**

**Generic name**	**Quebec generic code**
Clonidine	10751
Methyldopa	6136
Hydralazine	4524
Minoxidil	41564
Doxazosine	45625
Prazosin	37742
Terazosin	45520
Acebutolol	45463
Atenolol	43670, 46325,46315
Bisoprolol	47355
Carvedilol	47199, 46319
Labetalol	45243
Metoprolol	38275, 46763, 46780
Nadolol	40563
Oxprenolol	42162
Pindolol	39016
Pindolol-HCTZ	45408
Propranolol	8229
Sotalol	44866
Timolol	38314
Amlodipine	47006
Amlodipine/Atorvastatine	47609
Felodipine	45624
Nifedipine	42708, 46388, 46469
Nifedipine-AAS	47751
Nimodipine	45532
Diltiazem	43228, 47247
Verapamil	40550, 46573
Verapamil-Trandolapril	47440
Benazepril	47049
Captopril	42071
Cilazapril	47056
Cilazapril-HCTZ	47320
Enalapril	45476
Enalapril-HCTZ	45572
Fosinopril	47002
Lisinopril	45576
Lisinopril-HCTZ	47040
Perindopril	47117, 46258
Perindopril-Indapamide	47449
Quinapril	45629
Quinapril-HCTZ	47301
Ramipril	47079, 46216
Ramipril-HCTZ	47655
Trandolapril	47250
Trandolapril/Verapamil	47440
Candesartan	46529, 47309
Candesartan-HCTZ	46760, 47412
Eprosartan	47389
Eprosartan-HCTZ	47534, 47532
Irbesartan	46459 - 47282
Irbesartan-HCTZ	47354
Losartan	47135, 46284, 46441
Losartan-HCTZ	47207
Olmesartan medoxomil	47763
Olmesartan medoxomil-HCTZ	47764
Telmisartan	47333, 46587
Telmisartan-HCTZ	47413
Telmisartan-Amlodipine	47889
Valsartan	46418, 47259
Valsartan-HCTZ	47369
Spironolactone	9100, 46572
Amiloride	41759
Amiloride-HCTZ	41772
Hydrochlorothiazide	4537
Chlorthalidone	1976
Indapamide	43397
Metolazone	19440
Amiloride-HCTZ	41772
Spironolactone-HCTZ	38158
Triamterene-HCTZ	38197
Triamterene	9763
Aliskirene	47706
Aliskirene-HCTZ	47823
**Excluding the medications on the following formulation:**	
** Formulation**	**Code**
Ophthalmic powder	1479
Ophthalmic ointment	1624
Ophthalmic solution	2204
Ophthalmic and optic solution	2233
Ophthalmic suspension	2784
Ophthalmic and optic suspension	2813
Ophthalmic irrigation solution	3480
Ophthalmic gel	5365
Ophthalmic gel solution	5599

#### Diabetes mellitus

- ICD-9 codes: 250.0–250.9, 271.4 and 790.2
- ICD-10 codes: E10-E14 and R73.0
- Medication generic codes:

**Table:**

**Generic name**	**Quebec generic code**
Metformin	5824, 47208
Glucagon	4238
Chlorpropamide	1937
Glyburid	4264
Tolbutamide	9672, 15184
Gliclazide	46056, 47329
Glimepiride	46799, 47427
Acarbose	46300, 47151
Pioglitazone	46678, 47392
Rosiglitazone	47371, 46642
Rosiglitazone/Metformin	46862
Rosiglitazone/Glimepiride	47652
Nateglinide	46810
Repaglinide	47357, 46568
Saxagliptin	47817
Sitagliptin	47715
Sitagliptin/Metformin	47807, 47832
Alogliptin	48018
Alogliptin/Metformin	48017
Linagliptin	47881
Linagliptin/Metformin	47965
Insulin aspart/insulin aspart protamine	47615
Insulin detemir	47586
Insulin glargine	47536
Insulin lispro/insulin lispro protamine	47426
Insulin globine zinc	4823
Insulin sulfate	4888
Insulin crystal zinc (porc)	18296
Insulin protamine zinc (beef)	18309
Insulin protamine zinc (porc)	18322
Insulin isophane (porc)	18335
Insulin isophane (beef)	18348
Insulin slow release (beef and porc)	39120
Insulin isophane (beef and porc)	39133
Insulin protamine zinc (beef and porc)	39146
Insulin semi-slow release (beef and porc)	39159
Insulin ultra-slow release (beef and porc)	39172
Insulin crystal zinc (beef and porc)	39185
Insulin isophane (beef)*	39458
Insulin protamine zinc (beef)*	39484
Insulin protamine zinc (porc)*	39497
Insulin crystal zinc (beef)*	39523, 43735
Insulin slow release (porc)	41655
Insulin crystal zinc (porc)/ insulin isophane (porc)	43033
Insulin isophane semi-biosynthetic of human sequence	44151
Insulin slow release semi-biosynthetic of human sequence	44476
Insulin crystal zinc semi-biosynthetic of human sequence	44502
Insulin ultraslow release semi-biosynthetic of human sequence	44996
Insulins isophane and crystal zinc semi-biosynthetic of human sequence	45405
Insulin slow release biosynthetic of human sequence	45415
Insulin ultraslow release biosynthetic of human sequence	45483
Insulins isophane and crystal zinc biosynthetic of human sequence	45511
Insulins crystal zinc and isophane semi-biosynthetic of human sequence	45534
Insulin crystal zinc (beef and porc)	46536
Insulin isophane (beef and porc)	46537
Insulin slow release (beef and porc)	46538
Insulin isophane(human)/ insulin injectable(human)	46592
Insulin isophane (human)	46602
Insulin injectable (human)	46603
Insulin lispro/insulin isophane (human)	46607
Insulin crystal zinc (porc)	47004
Insulin lispro/ insulin lispro protamine	47426

#### Asthma

- ICD-9 codes: 493.0, 493.1, 493.3, 493.4, 493.5, 493.6, 493.7, 493.8 and 493.9
- ICD-10 codes: J45.0, J45.1, J45.8 and J45.9
- Medication generic codes:

For code with a * use only the following formulations:

**Table:**

**Formulation**	**Code**
Powder aerosol	1305
Powder aerosol with applicator	1334
Aerosol solution	1856
Aerosol solution with applicator	1885
Solution for Inhalation	1972
Suspension aerosol	2610
Suspension aerosol with applicator	2639
Inhalation powder with applicator	5563
Inhalation powder	5564
Oral spray	5584
Gel	5619
Powder for solution for inhalation	5634

#### Depression and anxiety disorders

- ICD-9 codes: 296, 309, 311, 300.0, 300.4
- ICD-10 codes: F30, F31, F32, F33, F34, F38, F39, F40, F41, F43.
- Medication generic codes:

**Table:**

**Generic name**	**Quebec generic code**
Citalopram	46543, 47317
Escitalopram	47553
Fluoxetine	45504
Fluvoxamine	45633
Paroxetine	46164, 47061
Sertraline	45630
Viibryd	NA
Desvenlafaxine	NA
Duloxetine	47714
Milnacipran	NA
Venlafaxine	46244, 47118
Isocarboxazid	5018
Phenelzine	7280
Tranylcypromine	9698
Amitriptyline	442, 429, 46836
Amoxapine	43696
Clomipramine	14781
Desipramine	2522
Doxepin	3198
Imipramine	4784
Maprotiline	37443
Nortriptyline	46835, 6578
Protriptyline	8294
Trimipramine	9906
Bupropion	46435, 47285
Buspirone	45609
Maprotiline	37443
Mirtazapine	46744, 47408
Reboxetine	NA
Trazodone	43137
Vilazodone	NA
Moclobemide	46427, 47005
L-tryptophane	42058
Nefazodone	46235, 47093

#### Thyroid disorders

- ICD-9 codes: 242.0-242.9, 244.0-244.9, 245.0-245.9, 246.1-246.9
- ICD-10 codes: E01, E02, E03, E05, E06 and E07
- Medication generic codes:

**Table:**

**Generic name**	**Quebec generic code**
Levothyroxine sodium	5252, 46574
Liothyronine sodium/levothyroxine sodium	33842
Liothyronine sodium	5317, 46457, 46474
Methimazole	40836
Propylthiouracil	8242

## Abbreviations

1DG: First day of gestation
aOR: Adjusted odd ratio
CI: Confidence interval
CTD: Connective tissue diseases
DMARDs: Disease-modifying antirheumatic drugs
GP: General practitioner
IBD: Inflammatory bowel disease
ICD-9: International Classification of Diseases Ninth Revision
ICD-10: International Classification of Diseases Tenth Revision
OR: Odd ratio
QPC: Quebec Pregnancy Cohort
RA: Rheumatoid arthritis
RAMQ: Régie de l’assurance maladie du Québec
SLE: Systemic lupus erythematosus
SpA: Spondyloarthropathies
